# Awareness and attitudes toward corneal donation among applicants and staff of a driver, vehicle and licensing authority (DVLA) in Ghana

**DOI:** 10.1186/s12886-019-1231-x

**Published:** 2019-11-12

**Authors:** Seth Lartey, Ellen K. Antwi-Adjei, Solomon Agyapong, Abdul-Kabir Mohammed, Derrick N. O. Mensah, Edward S. Genego

**Affiliations:** 1 Department of Eye, Ear, Nose and Throat. School of Medical Science. College of Health Sciences, KNUST, Kumasi, Ghana; 2 Department of Optometry and Visual Sciences, Faculty of Biosciences, College of Science Kwame Nkrumah University of Science and Technology, Kumasi, Ghana; 3 Consultant ophthalmic surgeon, Komfo Anokye Teaching Hospital, Kumasi, Ghana

**Keywords:** Knowledge, Willingness, Unwillingness, Cornea, DVLA

## Abstract

**Background:**

Corneal transplantations are surgeries performed for irreparable corneal diseases and damage. However, there is a gap between the number of potential recipients and the number of donor corneas available. The main aim of the study was to determine the awareness and attitudes toward corneal donation among applicants and staff of DVLA, Kumasi-Ghana.

**Methods:**

A descriptive cross-sectional study was conducted. One hundred participants were selected using convenient sampling method. A structured questionnaire was used to elicit responses from participants concerning awareness and attitudes toward corneal transplant.

**Results:**

The mean ± SD age of the participants was 32.05 ± 11.48 years and age range, 18-67 years. Males were 66% whilst females constituted 34%. 32.7% of the participants were aware of corneal donation. Majority of the participants were Christians (83.1%) and Singles (63%). Television was the source of information with the highest preponderance (49.4%). 67.3% were willing to donate their corneas after death. 63.9% were willing to indicate their donor statuses on drivers’ license form which had a significant association with willingness to donate cornea after death (*p* < 0.05, _*x*_^2^ = 12.187).

**Conclusion:**

There is a poor level of awareness (32.7%) of transplant and donation amongst the study population but a good level of willingness to donate organs (67%). Consent via driving license would seem to be a good potential mode of obtaining consent to supplement the harvesting of adequate tissues for transplant if adequate awareness is created.

## Background

Corneal transplantation involves the removal of a diseased or damage cornea and replacing it with a healthy donated cornea. However, one of the major barriers to corneal transplantation is low consent rate and unavailability of donors inducing a huge gap between the number of potential recipients and the number of donors available [1, 2].

Even though corneal transplantation is the most common type of transplant surgery worldwide, in many sub-Saharan African countries where the need is greatest, transplant services are not available [3].

Factors that affect unwillingness to donate include age, educational level, knowledge about donation, religious beliefs, associated health problems, objection from family members whilst factors that favor donation are the desire to help others and gender [4, 5].

There is limited literature on the knowledge and awareness level of Ghanaians about corneal transplantation. In Ghana corneal transplant is not routinely performed as there are no corneal banks available. Also, currently there is no legislature in Ghana regulating organ and tissue donation. It is unknown which type of organ donation system would work best in our environment. This makes it difficult to plan the best kind of National Cornea Donation program to adopt for a corneal transplant service.

The aim of this study was to determine awareness and attitudes towards corneal transplants among applicants and staff of DVLA and to also determine willingness to indicate their donor statuses on drivers’ license forms. This information would help in the planning and determination of the best donation systems to adopt.

## Methods

The study was a descriptive cross-sectional survey of drivers’ license applicants and staff of DVLA, Kumasi, Ghana. Hundred participants with age ≥ 18 years were recruited using convenience sampling method.

Data on demographics, socioeconomic status, knowledge corneal transplants, willingness to donate corneal after death, willingness to indicate donor status on drivers were collected using a structured questionnaire. Statistical Package for Social Sciences v22.0 was used for the analysis. *P*-values were obtained for the significant associations (*p* < 0.05). Chi- Square was used to assess associations between categorical variables.

## Results

### Socio-demographic characteristics

The study population consisted of 100 participants with mean age, 32.05 ± 11.48 years and age range, 18-67 years. Out of the sample, 66% were males and 34% were females. The ages of males ranged from 20 to 67 years with mean ± (SD), 33.98 ± 12.47 years and that of females ranged from 18 to 54 years with mean ± (SD) age, 28.29 ± 8.19 years (Table 1).

**Table 1:** Distribution of socio-demographic characteristics (*N* = 100)

Variable	Freq	(%)
Gender (*N* = 100)		
Male	66	66
Female	44	44
Age-Range/yrs. (*N* = 100)		
≤ 20	3	3
21–30	60	60
31–40	20	20
41–50	7	7
51–60	7	7
≥ 60	3	3
Marital Status (*N* = 100)		
Single	63	63
Married	32	32
Divorced	2	2
Widowed	3	3
Religion (*N* = 98)		
Christian	82	83.7
Muslim	15	15.3
Traditionalist	1	1
Educational Level(*N* = 100)		
No Formal Education	3	3
Elementary	6	6
Junior High	12	12
Senior High	12	12
Vocational/ Commercial	5	5
Training	9	9
Polytechnic	9	9
University	44	44
Residence(*N* = 96)		
Rural	27	28.1
Urban	69	71.9

*N* Number of participants, *Freq* Frequency of participants, *%* Percentage of participants, Variable-socio-demographic characteristic

### Sources of knowledge of transplant

**Fig. 1:**
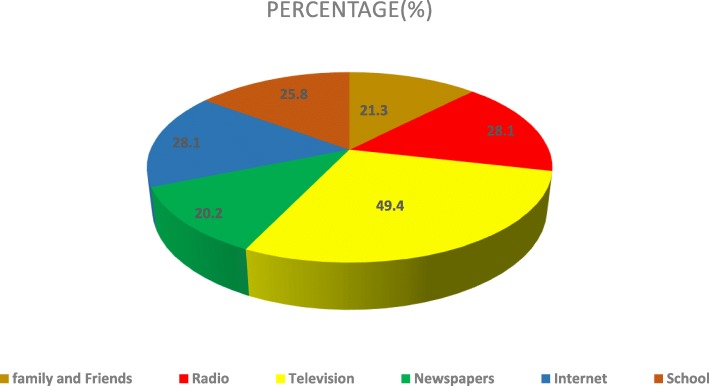
Sources of knowledge of transplant

Television was the main source of knowledge (49.4%) and Newspaper the least (20.2%) (Fig. 1).

### Knowledge and awareness of corneal donation for transplantation

Out of the 100 participants, 98% responded to the awareness of corneal transplant section out of which 32.7% of the respondents were aware of it.

52.94% of the female respondents knew about corneal transplant against 21.88% of males with a statistically significant association (*p = 0.002*, _*x*_^2^ = 9.745)

There was statistically significant association between awareness of corneal transplant and Ethnicity (*p = 0.018*, _*x*_^2^ = 14.822) and level of education (*p = 0.001*, _*x*_^2^ = 26.029). The association between knowledge of corneal transplant and occupation was strong (*p = 0.000*, _*x*_^2^ = 24.254) (Table 2.

**Table 2:** Association between knowledge of Transplant and Socio-demographic characteristics

Variable	Knowledge of corneal transplant		
	Freq(%)	_*x*_^2^	*P-Value*
Gender			
Male	14 (21.88)	9.745	0.002^*^
Female	16 (52.94)		
Ethnicity			
Akans	17 (26.98)		
Ga/ Adangme	6 (75)		
Ewe	3 (37.50)	15.294	0.018^*^
Guans	0 (0)		
Nzema	0 (0)		
Other	6 (60)		
Educational Level			
No formal Education	0 (0)		
Elementary	1 (20)		
Junior High	0 (0)	25.151	0.001^*^
Senior High	0 (0)		
Vocational	2 (40)		
Training	4 (44.44)		
Polytechnic	1 (11.11)		
University	24 (54.54)		
Residence			
Rural	6 (23.08)	1.925	*0.165*
Urban	26 (38.24)		
Marital Status			
Single	25 (39.68)	5.000	*0.172*
Married	6 (20.69)		
Divorced	1 (50)		
Widowed	0 (0)		
Age-Range (years)			
≤ 20	1 (33.33)	4.938	*0.424*
21–30	24 (40)		
31–40	5 (26.31)		
41–50	1 (14.29)		
51–60	1 (14.29)		
≥ 60	0 (0)		
Religion			
Christian	26 (32.10)	2.061	*0.357*
Muslim	5 (25)		
Traditionalist	1 (100)		

*significant at *p < 0.05*, _*x*_^*2*^ Chi-square value, *Freq* Frequency,Variable-socio demographic characteristics, *%* 01Percentage

### Attitudes toward corneal donation

**Fig. 2:**
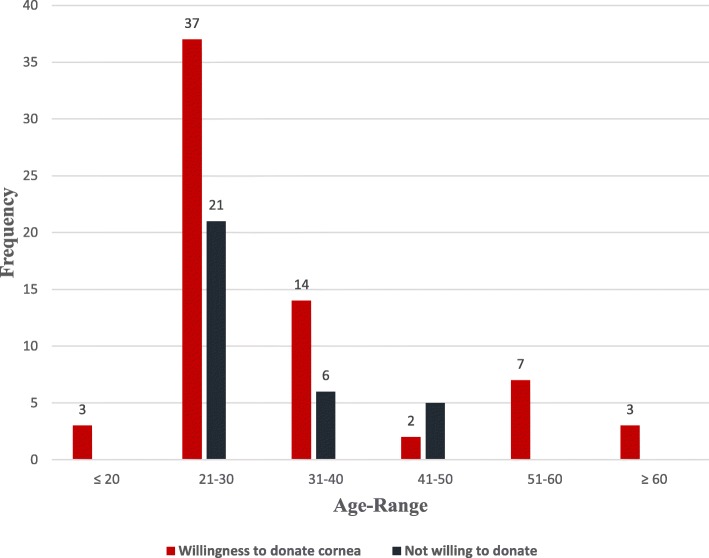
Willingness and Unwillingness to donate cornea after death mateched with Age-range

Out of the respondents to willingness to donate eyes (cornea) section, 67.3% were willing to donate their corneas after death.Age range was a relevant predictor of willingness to donate cornea after death (*p = 0.043*) with the age-range (≤ 20 years, 51-60 years and ≥ 60 years) with highest willingness to donate (Fig. 2).All the 32 participants who were aware of corneal transplant answered questions on corneal donation. Out of the respondents, 28(87.5%) reported that corneal donation is done surgically whereas 1(3.1%) indicated that it is done medically (using drugs) and 3(9.4%) reported that it can either be done surgically or medically (Table 3).

**Table 3:** Knowledge of Corneal Donation

Knowledge of Eye Donation	Freq (%)
Knowledge of Eye Donation (*N* = 32)	
It is giving the whole eye to someone with diseased eye	1 (3.1)
It is giving part of the eye (cornea) to others with diseased eye	12 (37.5)
It can be done to replace either part or whole eye	16 (50.0)
Do not know	3 (9.4)
Who can donate his Eye?(*N* = 32)	
Anyone	10 (31.3)
Only Adults	18 (56.3)
The aged	1 (3.1)
Do not Know	3 (9.4)
Victims of which death can donate their eyes (*N* = 32)	
Road Traffic accidents	7 (21.9)
Death through Diseases	4 (12.5)
Death natural causes	3 (9.4)
All types of Death	12 (37.5)
Do not Know	6 (18.50)

*Freq* Frequency of respondents, *%* Percentage

26% of the study participants answered the part that asked about reasons for not donating. Out of the respondents, 7.7% reported that, it was against their religious belief, 11.5% indicated their families were not in support, 26% did not have any reason and 43.2% did not have much knowledge about organ or corneal donation. On the reason for donating, 67.1% reported it was out of love for humanity; 20% indicated it was their religious obligation; 51.4 opined they were donating to give others the chance for a better life whilst 14.3% indicated they will only donate if they will take money (Table 4).

**Table 4:** Reasons for Donating and Decision Making

Reasons for donating and Decision making	Freq(%)
Reasons for Donating (multiple responses, *N* = 70)	
Love for humanity	47 (67.1)
My Religious Obligation	14 (20)
Give others the chance for a better life	36 (51.4)
If I will take money	10 (14.3)
Decision Making (Multiple responses)	
Myself (*N* = 97)	64 (66)
Family (*N* = 97)	28 (29.2)
Spouse (*N* = 96)	19 (19.6)
Children (*N* = 97)	27 (27.8)
Should You be paid for donating?(*N* = 98)	
Yes	29 (30.2)
No	67 (69.8)
Should Your surviving family be paid?(*N* = 94)	
Yes	39 (41.5)
No	55 (58.5)

*N* Number of respondents, *Freq* Frequency of respondents, *%* Percentage of respondents

Participant indicated their reservations about donations in Ghana (multiple responses).The majority (50.5%) indicated that they do not have much information about donation. Most of the respondents (36.6%) also indicated they do not trust the Ghana health system and 18.3% reported that their families will not agree.Less than 50% of the respondents knew that one can donate his organ when alive or dead (Table 5).

**Table 5:** Concerns and Reservations about Donation and Transplant in Ghana

Concerns and Reservations of Donation	Freq (%)
Concerns of Donation (multiple responses)	
I do not have enough information about organ donation	49 (50.5)
I do not like the thought of people taking out my body parts	18 (18.6)
I think my body would be grossly disfigured when taken out	19 (19.6)
I want all my body parts to be intact during my funeral	14 (14.4)
It is against my religious belief	13 (13.4)
It is a taboo in my culture	11 (11)
I am uncomfortable with this topic and don’t want to discuss it	11 (11)
Reservations	
I do not trust the Ghana Health system	34 (36.6)
I’m afraid my body parts would be used for rituals	7 (7.5)
I’m afraid my body parts may be sold for profit	6 (6.5)
I’m afraid Doctors may not do much to my life when I’m sick because they want my body parts	6 (6.5)
My family will not agree	17 (18.3)
I have no reservation	22 (23.7)
When can one donate body parts?	
Whilst Alive	36 (40.4)
After Death	7 (7.9)
Both	40 (44.9)
Do not know	6 (6.7)
Is Organ and Corneal Donation Urgent in Ghana?	
Yes	62 (76.5)
No	19 (23.5)

*Freq* Frequency of respondents, *%* Percentage of respondents, *Reservations* Reservations toward donation

### Willingness to indicate donor status on drivers’ license form

97% of the sample stated whether they were willing to indicate their donor statuses when going for drivers’ license. Out of the respondents, 63.9% of them were willing to indicate their donor statuses on the Drivers’ License form when going for them. There was statistically significant association between willingness to indicate Donor status on Drivers’ License form and willingness to willingness to donate cornea after death (*p = 0.000*, _*x*_^2^ = 12.187) (Table 6).

**Table 6:** Association between willingness to donate cornea and willingness to indicate donor status on drivers’ license form

Willingness to Donate	Willingness to indicate Donor Status on License Form		
	Freq (%)	_*x*_^2^	*P*-Value
Cornea	49 (75.54)	12.187	0.000^*^

^*^significant at *p < 0.05, Freq* Frequency of respondents, _*x*_^*2*^ Chi Square value

## Discussion

Awareness level of corneal transplant among the respondents in this study was low (32.7%). A similar result was found in Medina, Saudi Arabia where 35.8% of the study population were aware of corneal transplant [6] This contrast very much with Edwin and Raja (2000) who reported awareness regarding transplantation of eye, 88% [7]. It is also lower than the research by Vijayhhmahantesh et al. where 95.6% out of the 1052 participants knew about corneal transplant [8] perhaps because corneal transplant is routinely performed in that country, people have become more aware.

Out of the respondents, 67.3% were willing to donate their corneas after death. A relatively lower rate of donation was found in a study conducted in Saudi Arabia where 21.1% were willing to donate their eyes [6]. Willingness to donate cornea had a significant association with age range (*p = 0.043*, _*x*_^2^ = 11.486). Old and very young people are more willing to donate than the mid-aged people.

The majority of the participants wanted to donate mainly because of altruistic and religious values; 67.1% reported that they will donate because of love for humanity, 20% opined that it was their religious obligation, 51.4% reported they will donate to give others the chance for a better life.

Less than 50% which is 44.9% out of the respondents knew that a body can be donated whilst alive or dead which is lower than the 53.1% reported by Sandeep et al., (2017) [9].

Out of the people who knew about corneal transplant, 37.5% knew that corneal donation is done by giving part (cornea) of the eye to others with diseased part (cornea). The results indicated that even the few people who had heard about cornea transplant did not know much about the procedure itself.

31.3% reported that anyone can donate his eyes. This show the lack of accurate knowledge of the procedure. 37.5% of the respondents reported that victims of every death qualify for eye donation provided the cornea is healthy.

These low rates of accurate knowledge given about the procedure by the respondents, indicates the need for awareness creation about cornea transplant.

This study revealed that 50.5% of the respondents did not have enough information about organ donation;18.6% did not like the thought of people taking away their body parts; 19.6% believed their bodies would be grossly disfigured when their body parts are taken out; 14.4% wanted all their body parts to be intact during the funerals; 13.4% reported that it was against their religious beliefs; 11% opined it was a taboo in their culture and 11% were not comfortable discussing the topic and did not want to discuss it. Most respondents were concerned about the state of their bodies after death because of the common belief amongst Africans that one must return to the ancestral world with a complete body in order to have a peaceful rest otherwise the person’s spirit will warn and haunt the living for vengeance. A similar study conducted by Pike et al. (1993) revealed that what prevented majority of the people from donating was lack of knowledge about organ transplantation which correlates with our findings [10]. A study in Pakistan had similar reasons where participants reported that “the body belongs to God and God gave it to man and no man can give it to another person” [11].

Out of the respondents, 63.9% were willing to indicate their donor status on drivers’ license form. There was a significant association between willingness to indicate donor status on drivers’ License form and willingness to donate eye (_*x*_^2^ = 12.187, *p < 0.05*).

### Limitation of the study

This study was carried out at only one DVLA Centre.

## Conclusion

This study has revealed low level of awareness of cornea transplant among participants (32.7%). More than half of the study population reported that they were willing to donate their organs and corneas (67%.). The main reason for unwillingness to donate was lack of knowledge about donation to make the decision. More than half of the participants stated that they were willing to indicate on the Drivers’ License form their donor status. There was a strong relationship between willingness to indicate donor status on Drivers’ license form and willingness to donate cornea. Consent via driving license would seem to be a good potential mode of obtaining a donor pool to supplement the harvesting of adequate tissues for transplant if adequate awareness is created.

## Data Availability

The datasets used and analysed during the current study are available from the corresponding author on reasonable request.

## Abbreviations

DVLA: Driver Vehicle and Licensing Authority
SD: Standard Deviation
